# Simvastatin Modulates Astrocyte and Microglial Reactivity and Aggressive Behavior After Chronic Sleep Deprivation in Rats

**DOI:** 10.1002/jnr.70160

**Published:** 2026-11-01

**Authors:** Higor Hernany Dias, Kevin Silva Müller, Gabriela Larissa Lima‐da‐Silva, Stephane da Silva Alves, Tatiane de Camargo Martins, Pedro Tadao Hamamoto Filho, José de Anchieta de Castro e Horta‐Júnior

**Affiliations:** ^1^ Medical School, Botucatu (FMB), São Paulo State University (UNESP) Botucatu SP Brazil; ^2^ Laboratory of Nerve Regeneration Institute of Biology (IB), State University of Campinas (UNICAMP) Campinas SP Brazil; ^3^ Department of Anatomy Institute of Biosciences of Botucatu (IBB), São Paulo State University (UNESP) Botucatu SP Brazil

**Keywords:** amygdala, glial cells, hippocampus, sleep fragmentation, statin

## Abstract

Chronic sleep deprivation (SD) disrupts central nervous system homeostasis, promoting behavioral alterations and glial remodeling within limbic structures. Although simvastatin exhibits pleiotropic effects, its influence on astrocytic and microglial responses during prolonged sleep loss remains poorly understood. This study investigated the effects of SD and simvastatin on behavior, systemic inflammatory mediators, and glial reactivity in the dorsal hippocampus (HPC) and amygdala (AMY). Sixty male Wistar rats (~75 days old; *n* = 15/group) were assigned to Control (CG), Sleep Deprived (SD), Simvastatin 4.2 mg/kg (SIM4.2), or Simvastatin 20 mg/kg (SIM20) groups. Animals underwent the modified multiple platform method (18 h/day) for 45 consecutive days, with daily oral simvastatin administration. Social behavior was assessed using the resident‐intruder test, circulating cytokines by ELISA, and glial responses by GFAP and IBA‐1 immunohistochemistry, including microglial morphological classification (Types I–III). SD increased aggressive/reactive behavior and astrocytic and microglial reactivity in the dorsal HPC, accompanied by a shift from homeostatic (Type I) toward reactive (Types II and III) microglial phenotypes. Simvastatin attenuated these glial alterations, restoring a more homeostatic microglial profile and increasing non‐social exploratory behavior. In contrast, glial alterations in the AMY were comparatively limited, indicating region‐specific susceptibility to SD. Peripheral cytokine analysis revealed selective immunomodulatory effects, including reduced TGF‐β and increased IL‐8 in SIM20, whereas TNF‐α and IL‐6 remained unchanged. Overall, simvastatin modulated behavioral, systemic inflammatory, and glial responses to SD, supporting its pleiotropic effects and highlighting region‐specific glial remodeling as a potential target for mitigating the consequences of chronic sleep loss.

## Significance Statement

Chronic sleep deprivation severely disrupts central nervous system homeostasis and triggers neuroinflammation, yet targeted pharmacological strategies remain limited. This study demonstrates that prolonged sleep loss selectively drives astrocytic and microglial reactivity in the dorsal hippocampus—shifting microglia toward reactive phenotypes and increasing aggressive behavior—while leaving the amygdala relatively unaffected. Daily administration of simvastatin attenuates these hippocampal glial alterations, restores homeostatic microglial morphology, and improves behavioral outcomes. These findings highlight region‐specific neuroglial vulnerability to chronic sleep debt and demonstrate the pleiotropic neuroprotective potential of simvastatin in mitigating sleep‐loss‐induced central pathology.

## Graphical abstract

Simvastatin modulates chronic sleep deprivation‐induced glial remodeling and behavioral responses.

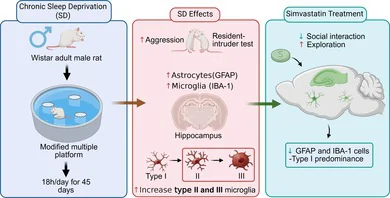

## 1 Introduction

Chronic sleep deprivation (SD) is a growing problem in society, which is a biologically significant stress factor that affects the homeostasis of the central nervous system (CNS), contributing to memory problems, attention deficit, and other disorders, including depression and anxiety (Bishir et al. 2020; Goldstein‐Piekarski et al. 2015; Hyndych et al. 2025; Krause et al. 2017; Thompson et al. 2022). Sleep deprivation has been associated with changes in memory consolidation, emotional regulation, and social behavior, processes predominantly mediated by the limbic system (Goldstein and Walker 2014; T. Kim et al. 2022). Among these structures, the hippocampus (HPC) and amygdala (AMY) play central and complementary roles in integrating cognitive and emotional information (Qasim et al. 2023; Šimić et al. 2021).

The HPC and AMY are key limbic structures involved in coordinating cognitive and emotional responses to stress (Anand and Dhikav 2012). The HPC is essential for memory formation, contextual processing, and adaptation to stress, with its dorsal portion classically associated with cognitive processes, as well as learning and spatial navigation (Fanselow and Dong 2010). Since the HPC is a structure related to neurogenesis and neuroplasticity, it ends up being especially vulnerable to stressful factors, such as sleep deprivation (Conrad 2008; Prince and Abel 2013). In contrast, the AMY primarily mediates emotional processing and defensive behaviors (M. J. Kim et al. 2011). The basolateral nucleus (BLA) of AMY is associated with emotional aspects of behavior, while the central nucleus (CeA) of AMY acts as an important output structure that regulates fear, anxiety, and aggression (AbuHasan et al. 2023; Haller 2018; Yang and Wang 2017). Functional connectivity between the HPC and AMY enables coordinated regulation of socioemotional behavior, making these regions particularly relevant for understanding the neural consequences of SD (Yang and Wang 2017).

Glial cells are key regulators of neural circuit stability and inflammatory signaling within these regions (Verkhratsky et al. 2019). Astrocytes perform essential functions in maintaining CNS homeostasis, such as maintaining blood–brain barrier (BBB) stability and providing metabolic support to neurons, whereas microglia actively monitor the brain microenvironment and respond to disturbances in the tissue, such as pathogen exposure (Allen and Barres 2009; Barres 2008; Jäkel and Dimou 2017; Liddelow et al. 2017). SD has also been associated with changes in glial reactivity and inflammatory mediators (Que et al. 2023).

Statins, such as simvastatin, have been associated with potential pleiotropic effects beyond cholesterol reduction, including modulation of inflammatory responses and glial reactivity (Alves 2021; Churchward and Todd 2014; Istvan and Deisenhofer 2001; Liao and Laufs 2005). Although simvastatin has been shown to attenuate astrocytic and microglial reactivity in models of neural injury and neuroinflammation (Bobermin et al. 2024; Chen et al. 2013; Jafari et al. 2021; Li et al. 2009; Vuu et al. 2023; Wu et al. 2010), its effects on SD‐induced glial responses within limbic circuits remain poorly characterized.

In the present study, we investigated astrocyte and microglial reactivity in the dorsal HPC and AMY (BLA and CeA nucleus) following SD and evaluated whether simvastatin modulates these cellular responses in association with alterations in social and aggressive behavior.

## 2 Methods

### 2.1 Animals and Experimental Design

This study was conducted in accordance with the ARRIVE guidelines (Percie du Sert et al. 2020). All animal procedures were conducted in accordance with the guidelines of the Brazilian National Council for the Control of Animal Experimentation (CONCEA) and were approved by the Institutional Animal Care and Use Committee (protocol no. 1414/2022) of São Paulo State University (UNESP), Botucatu, SP, Brazil. Although recent recommendations support the inclusion of females in biomedical research, sex differences in sleep deprivation models, particularly regarding sleep architecture and emotional and cognitive outcomes, remain inconsistent and have not been fully characterized in the literature (Q. Deng, Li, et al. 2024; Fernandes‐Santos et al. 2012). Therefore, male rats were used to promote greater experimental standardization, reduce biological variability, and enhance reproducibility (Beery and Zucker 2011; Prendergast et al. 2014). Furthermore, the resident‐intruder (RI) paradigm has been reported to produce more robust and consistent behavioral responses in males than in females (Koolhaas et al. 2013).

Sixty male Wistar rats ( *Rattus norvegicus* ) were obtained from the University of São Paulo breeding facility and housed under controlled environmental conditions, including a temperature of 22°C ± 2°C and a 12‐h light/dark cycle (lights on from 5:00 p.m. to 5:00 a.m.), with filtered water and Presence feed provided ad libitum. Animals were housed in pairs or trios in standard cages with environmental enrichment consisting of knots made from natural sisal rope. The rats were maintained in the animal facility of the Center for Toxicological Assistance (CEATOX), Institute of Biosciences of Botucatu (IBB), UNESP, until they reached 75 days of age (Figure 1). Rats were randomly assigned to four experimental groups (*n* = 15 per group): control (no intervention; CG), sleep deprivation (SD), and sleep deprivation treated with simvastatin at 4.2 mg/kg (SIM4.2) or 20 mg/kg (SIM20). The doses selected for this study were based on conversion of the average therapeutic dose in humans (40 mg/day of simvastatin) using the dose‐by‐factor method (Nair and Jacob 2016).

**FIGURE 1:**
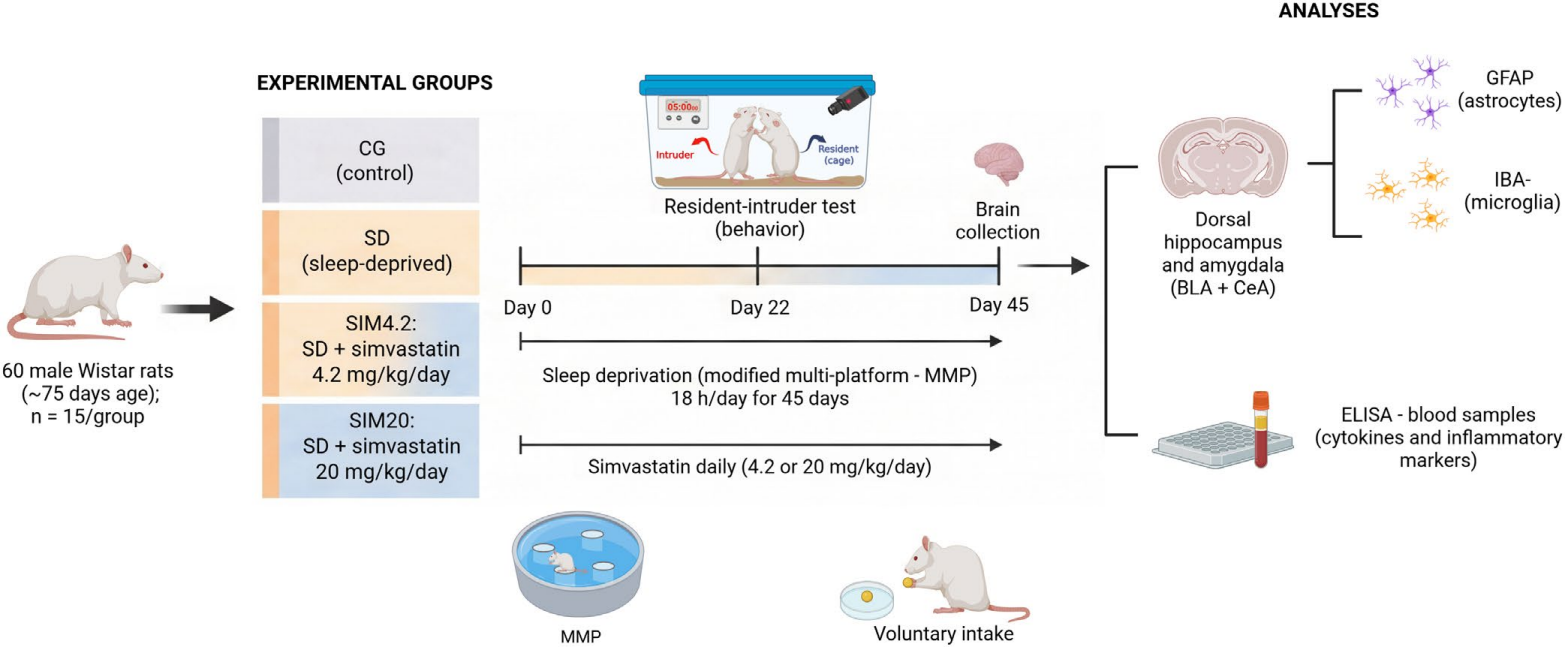
Experimental design.

### 2.2 Sleep Deprivation Protocol

To induce sleep deprivation, the modified multiple‐platform method was used. This method consists of a water tank containing small platforms at the bottom (Figure 1, Data S1). In this method, animals are placed in a tank containing multiple platforms surrounded by water. Over time, particularly at the onset of the paradoxical sleep period, the animals lose muscle tone and fall from the platforms into the water, thereby awakening from sleep (Machado et al. 2004). This method has been shown to be effective in completely suppressing REM sleep. Previous studies have demonstrated that animals housed in groups and in pairs or trios exhibit lower levels of stress when exposed to sleep deprivation (Machado et al. 2004). The procedure was performed in a temperature‐controlled room maintained at 22°C. Polypropylene boxes measuring 94 × 61 × 30 cm (internal dimensions) and 102 × 68 cm (external dimensions) were used. Each box contained 18 square acrylic platforms (6.5 cm in diameter) supported by polyvinyl chloride (PVC) pipe bases, 8 cm in height. The platforms were spaced 7 cm apart, and the boxes were filled with water to a depth of 3 cm, maintained at room temperature (25°C), approximately 1 cm below the platform surface (Zhang et al. 2018). The boxes were covered with an iron grid. Throughout the sleep deprivation period, the animals had free access to water and feed ad libitum. The sleep deprivation protocol was performed daily from 2:00 p.m. to 8:00 a.m. for 45 consecutive days (Machado et al. 2004). During the remaining period, the animals were allowed to sleep in their home cages and were housed in pairs or trios to minimize stress associated with social isolation.

### 2.3 Administration of Simvastatin

For each group, a mixture of commercial whipped cream powder (Chantilinho Mix) and deionized water was prepared daily and used as the vehicle. For the groups treated with simvastatin, the appropriate dose of simvastatin was added to the vehicle: SIM4.2 (4.2 mg/kg/day) or SIM20 (20 mg/kg/day). The dose was calculated based on the group's mean body weight measured on the previous day. To ensure uniform distribution of the drug, the required amount of simvastatin powder (C_25_H_38_O_5_; molecular weight [MW] 418.57; CAS No. 79902‐63‐9) was mixed with the whipped cream powder and homogenized using a vortex mixer in a Falcon tube. The amounts of simvastatin and deionized water were calculated and added to the vehicle to form a firm mass which was divided into individual daily portions of 250 mg using an analytical balance and manually shaped into small pellets (Data S2). The animals were habituated to voluntarily consume one 250‐mg pellet, once daily at 8:00 a.m. for 45 consecutive days. The CG and SD groups received the same 250‐mg portion containing only whipped cream powder and purified water.

### 2.4 Behavioral Test: Resident‐Intruder Paradigm (RI)

The resident‐intruder (RI) test is used to assess social interaction and aggressive and defensive responses in rodents under controlled conditions and has been widely used to evaluate the functional responses of the AMY and HPC (Koolhaas et al. 2013). In this test, two adult rats with no prior contact are placed in the same cage, with one rat (the intruder) introduced into the cage of the other rat (the resident) to allow social interaction. A common application of this test is to assess social interactions between rats. The resident rat establishes territory in its home cage, and the presence of an unfamiliar rat may elicit aggressive and potentially stressful behaviors (Kraak 2012).

Fifteen animals per group were included in the behavioral assessments, of which eight per group were subsequently used for blood collection by decapitation for ELISA analysis. The behavioral test was performed 22 days after the initiation of treatment. Before the test, the animals were transferred from the animal facility to a separate room and allowed to acclimate for 1 h to minimize stress associated with the change in environment. Subsequently, the intruder rat was introduced into the resident rat's home cage. The animals were allowed to interact for 5 min, and the entire session was video‐recorded for subsequent behavioral analysis (Figure 1). The behavioral repertoire of the resident rats was assessed based on the following behaviors: aggressive/reactive interaction (move toward with biting), anogenital investigation, non‐anogenital social interaction, clinching, chasing, non‐social exploration, lateral threat, mounting, biting, and upright posture (Koolhaas et al. 2013).

### 2.5 Tissue Collection and Processing

After 45 days of treatment, animals were euthanized under deep anesthesia induced by intraperitoneal administration of ketamine (300 mg/kg), xylazine (30 mg/kg), and acepromazine (1 mg/kg). Adequate anesthetic depth was confirmed by the absence of corneal and withdrawal reflexes.

Six animals per group were transcardially perfused with 0.9% saline solution (30 mL/min), followed by freshly prepared 4% formaldehyde in 0.1 M phosphate buffer (pH 7.4) for 30 min. Body weight and naso‐anal length were recorded before perfusion. The brains were removed by dorsal craniotomy, weighed, and post‐fixed overnight at 4°C. Brains were sectioned into three coronal blocks at the levels of the pons and optic chiasm using a brain matrix and cryoprotected in 30% sucrose in phosphate buffer for at least 48 h. The block containing the HPC and AMY was frozen and coronally sectioned at 30 μm using a sliding microtome equipped with a freezing stage. Sections were stored in an antifreeze solution at −20°C until further processing.

### 2.6 Enzyme‐Linked Immunosorbent Assay (ELISA)

Blood samples were collected after euthanasia and stored at −80°C in a freezer at the Experimental Research Unit (UNIPEX), UNESP, Botucatu, Brazil. Interleukins (IL‐4, IL‐5, IL‐6, IL‐8, and IL‐10), TNF‐α, and TGF‐β were measured to determine the animals' systemic inflammatory profile. The assays were performed using serum samples from animals in the CG, SD, and SIM20 groups (CG, *n* = 7; SD, *n* = 7; SIM20, *n* = 8). Commercial ELISA kits specific for rats were used for IL‐4 (E‐EL‐R0014, Elabscience), IL‐5 (E‐EL‐R0558, Elabscience), IL‐6 (E‐EL‐R0015, Elabscience), IL‐8 (EK720269, AFG Scientific), IL‐10 (E‐EL‐R0016, Elabscience), TNF‐α (E‐EL‐R2856, Elabscience), and TGF‐β (E‐EL‐R0084, Elabscience). All samples were analyzed in duplicate, and the assays were performed strictly according to the manufacturers' instructions, from plate preparation through data acquisition using a microplate reader (Epoch 2, BioTek). Data were expressed as cytokine concentrations in picograms per milliliter (pg/mL).

### 2.7 Immunohistochemical Protocol for GFAP and IBA‐1

For morphological analysis of the dorsal HPC and AMY, six brains from each experimental group were randomly selected (*n* = 6/group; total *n* = 24). Two series of sections from each brain were processed: one for microglial labeling (IBA‐1) and the other for astrocytic labeling (GFAP). Sections were washed in 0.1 M phosphate‐buffered saline (PBS; pH 7.4), endogenous peroxidase activity was quenched with 3% hydrogen peroxide for 20 min, and nonspecific binding was blocked with 2% normal goat serum (S1000, Vector Laboratories) in 0.05 M Tris buffer containing 0.25% Triton X‐100 (TBS‐TX). Sections were then incubated for 24 h at 4°C under continuous agitation with either the primary anti‐IBA‐1 antibody (rabbit, #019‐19741, FUJIFILM/Wako, Osaka, Japan; 1:5000) or anti‐GFAP antibody (rabbit, #AB207165, Abcam, Cambridge, UK; 1:5000). After washing, sections were incubated with a biotinylated secondary antibody (BA‐9200, Vector Laboratories; 1:200) for 2 h at room temperature, followed by incubation with an avidin‐biotin‐peroxidase complex (Vectastain ABC Kit, PK‐4000; 1:200) for 2 h. Immunoreactivity was visualized using 3,3′‐diaminobenzidine tetrahydrochloride intensified with nickel ammonium sulfate (DAB‐Ni), yielding a stable black reaction product. Finally, sections were mounted on gelatin‐coated slides, coverslipped with Permount (Fisher Scientific), and prepared for microscopic analysis.

#### 2.7.1 Cell Counting and Image Analysis

Photomicrographs of sections processed for GFAP and IBA‐1 immunohistochemistry (*n* = 6/group) were acquired using an optical microscope (AxioPlan 2, Carl Zeiss) coupled to a digital camera (AxioCam HRc, Zeiss). The specific regions of the dorsal HPC analyzed included the CA1 and CA3 regions and the upper and lower portions of the dentate gyrus, as well as the basolateral (BLA) and central (CeA) nuclei of the AMY. Images were acquired at five anteroposterior levels, ranging from −3.8 to −2.6 mm relative to bregma, for analysis of the dorsal HPC (Figure 2), and at four anteroposterior levels, ranging from −2.9 to −2.0 mm relative to bregma, for analysis of the AMY (Figure 3) (Paxinos and Watson 2007).

**FIGURE 2:**
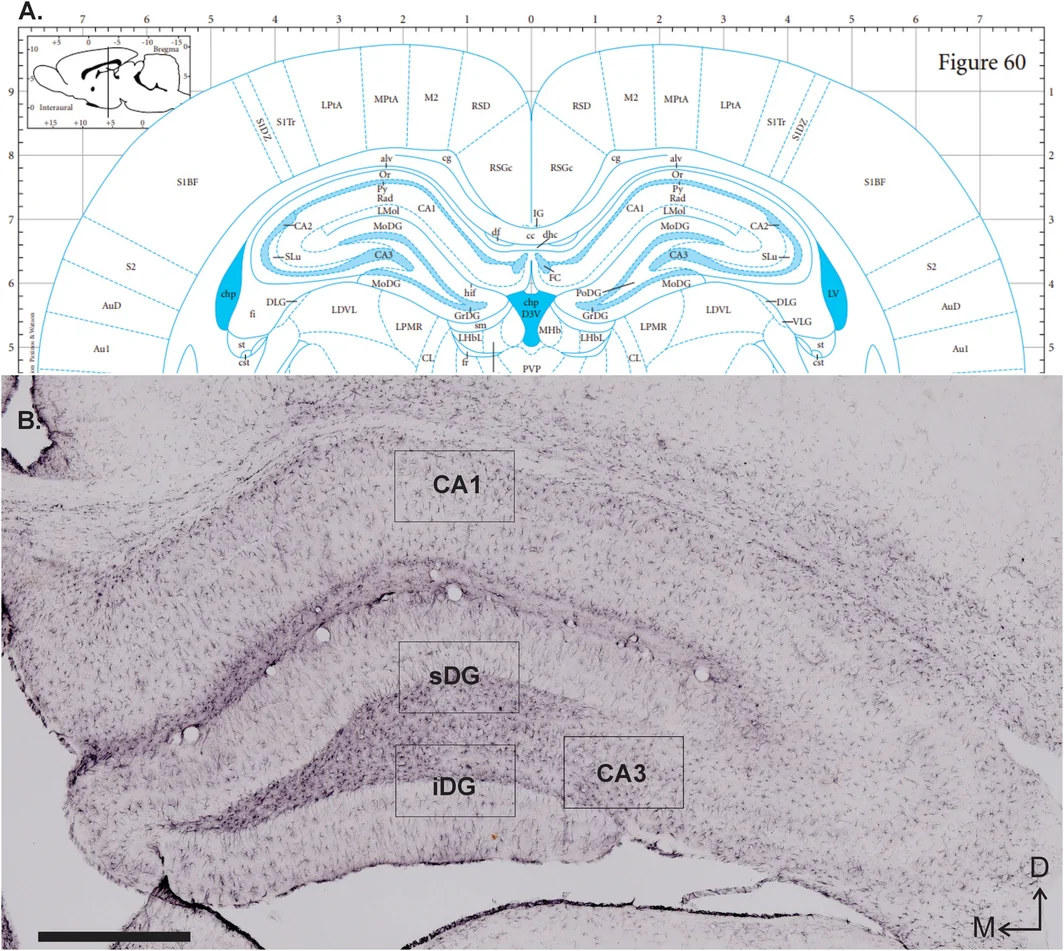
Dorsal hippocampus (HPC) and regions of interest (ROIs) used for cell counting analysis. (A) Schematic representation from the atlas of Paxinos and Watson (2007), at the level of bregma −3.20 mm. (B) Photomicrograph of a coronal section of the dorsal HPC following GFAP immunohistochemistry. The rectangular areas indicate the ROIs analyzed in the dorsal HPC: CA1, CA3, upper portion of the dentate gyrus (sDG), and lower portion of the dentate gyrus (iDG). Scale bar = 200 μm. Orientation arrows: D, dorsal; M, medial.

**FIGURE 3:**
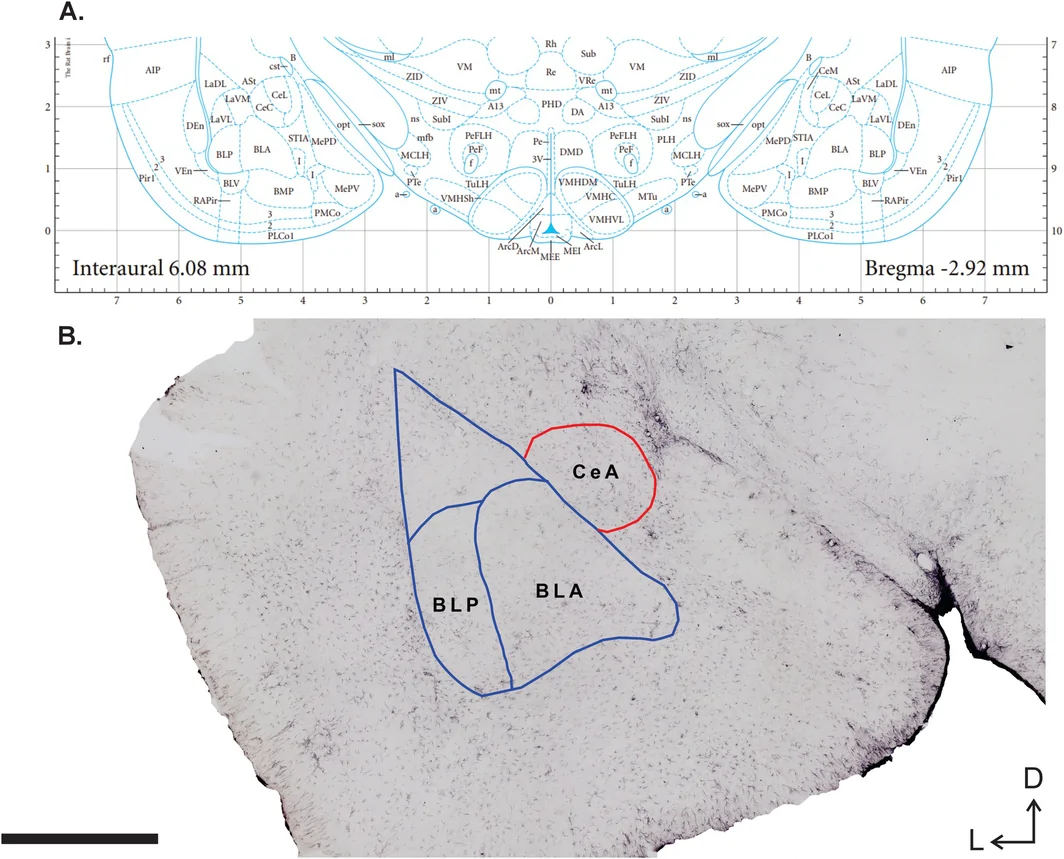
Amygdala (AMY) and delineation of the nuclei analyzed for cell counting. (A) Schematic representation from the atlas of Paxinos and Watson (2007), at the level of bregma −2.90 mm. (B) Photomicrograph of a coronal section of the AMY showing the delineation of the basolateral (BLA, blue line) and central (CeA, red line) nuclei, following GFAP immunohistochemistry. Scale bar = 200 μm. Orientation arrows: D, dorsal; L, lateral.

To evaluate IBA‐1 and GFAP immunoreactivity, a semiquantitative analysis was performed by counting immunoreactive cells using the open‐source software Fiji, version 2.17.0, with the Cell Counter plugin. Only cells with clearly visible nuclei and complete cellular processes were counted. The total number of immunoreactive cells in each region of interest (ROI) for each animal was calculated by summing the counts obtained across all analyzed levels.

Cell counting was performed within standardized ROIs measuring 500 × 300 μm for all HPC regions. The ROIs were positioned in the CA1, CA3, and dentate gyrus regions of the dorsal HPC, with the dentate gyrus divided into upper (sDG) and lower (iDG) portions (Figure 2). For the AMY, standardized ROIs measuring 400 × 400 μm were used at each of the four analyzed levels and positioned within the basolateral (BLA) and central (CeA) nuclei of the AMY.

#### 2.7.2 Microglial Morphological Classification

Microglial morphological profiling was performed using the same IBA‐1‐stained mosaic images used for cell counting (*n* = 5/group). Representative coronal sections from each animal were selected, with analysis focused on standardized regions of interest (ROIs) within the amygdala and hippocampal formation.

All IBA‐1‐positive (IBA‐1+) cells were classified into three distinct morphological phenotypes based on changes in somal morphology and process arborization (Diz‐Chaves et al. 2012; Muller et al. 2026).: Type I (surveillance/resting) cells exhibited small, delicate cell bodies with an extensive and highly ramified network of thin processes. Type II (transition/alert) cells exhibited moderately enlarged, rounded somata with thickened primary branches and a noticeable reduction in distal ramifications. Type III (hypertrophic/reactive) cells displayed prominently enlarged and intensely stained cell bodies with shortened, markedly thickened, and retracted processes, accompanied by a clear loss of fine secondary arborization.

For functional and categorical analyses, because fully amoeboid (Type IV) or degenerative (Type V) profiles were completely absent from the analyzed brain regions, the microglial population was dichotomized into two biologically distinct operational groups: homeostatic/resting microglia (Type I), representing the baseline physiological surveillance state, and reactive/transitioning microglia (Types II and III).

This binary categorization is supported by studies showing that acute or short‐term sleep loss can induce rapid morphological and transcriptional changes, characterized by a transition from highly complex, ramified structures toward less complex, activated profiles with localized somal enlargement, rather than a pronounced hyperinflammatory response (Bellesi et al. 2017; J. Kim et al. 2024; Liu et al. 2022). Consequently, this process may result in an increased proportion of intermediate Type II phenotypes rather than terminal macrophage‐like morphologies (Bellesi et al. 2017; Liu et al. 2022; Parhizkar et al. 2023). Furthermore, defining the hypertrophic Type III phenotype as the highest degree of microglial activation assessed in this experimental design is consistent with the low‐grade neuroinflammatory profile described in sleep‐restriction models (Manchanda et al. 2018).

### 2.8 Sample Size Calculation

For the microglial morphological classification, the sample size was determined using the formula proposed by Dell et al. (2002): *n* = 1 + 2C(s/d)^2^, where C = (zcrit + zpwr)^2^. To account for multiple pairwise comparisons among the four experimental groups (six possible pairwise comparisons), the significance level was adjusted to *α* = 0.0083 using the Bonferroni correction (zcrit = 2.638). Assuming a statistical power of 80% (1 − *β* = 0.80; zpwr = 0.842), a population standard deviation (s) of 0.20, and an expected magnitude of difference (d) of 0.50, the formula yielded a sample size of *n* = 4.88. This value was rounded up to five rats per group (total *N* = 20). For cell counting and image analysis, an additional 20% safety margin was included to account for potential tissue loss, fixation artifacts, or section folding during histological processing. This resulted in a final sample size of six animals per group (total *N* = 24), consistent with previous research conducted by our group (Balin et al. 2020).

For the behavioral tests, a higher standard deviation was anticipated because of the greater variability typically observed in behavioral measures (s = 0.33). Using the same formula, the estimated sample size was *n* = 11.55. Therefore, a minimum of 12 animals per group was required (Hyer et al. 2021). Considering that some animals could be excluded from the analysis for failing to meet the inclusion criteria (i.e., absence of disease or injury), an additional 20% safety margin was included, resulting in a final sample size of 15 animals per group (total *N* = 60).

### 2.9 Statistical Analysis

Quantitative variables were statistically compared and analyzed. Before conducting the analyses, the data were assessed for normality and homoscedasticity. Potential outliers were identified using the ROUT method (Motulsky and Brown 2006), with a *Q* value of 2%. Data that met both assumptions were analyzed using parametric tests, specifically one‐way analysis of variance (ANOVA) followed by Tukey's post hoc test. Data that did not meet the established assumptions were analyzed using nonparametric tests, specifically the Kruskal–Wallis test followed by Dunn's post hoc test. To investigate potential associations between glial reactivity and behavioral outcomes, Pearson correlation coefficients were calculated between the numbers of GFAP‐ and IBA‐1‐immunoreactive cells (total dorsal HPC, total AMY, BLA, and CeA) and behavioral variables recorded during the resident‐intruder test, including anogenital investigation, non‐anogenital social interaction, chasing, non‐social exploration, and aggressive/reactive interaction. Correlation results are reported as *r* and *p* values. Results are expressed as mean ± SEM for most analyses, except for immunohistochemical cell counting and morphological analyses, which are expressed as mean ± SD. All statistical analyses and graphical representations were performed using GraphPad Prism software, version 9.0.0. For all analyses, statistical significance was set at *α* = 0.05.

## 3 Results

### 3.1 Behavior Analysis: Resident‐Intruder Paradigm

Although these behaviors were included in the behavioral assessment, no overtly aggressive behaviors, such as clinching, lateral threat, mounting, or upright posture, were observed in any of the animals, as observed in Figure 4A. Regarding aggressive/reactive interactions, an increase was observed in the SD group compared with the CG group (*p* = 0.0235) (Figure 4B). In the SIM4.2 and SIM20 groups, decreases were observed in chasing, anogenital investigation, and non‐anogenital social interaction compared with the untreated groups (CG and SD) (Figure 4C,D,F). For chasing behavior, a reduction was observed in the SIM20 group compared with both the CG group (*p* = 0.0222) and the SD group (*p* = 0.0184) (Figure 4C). Regarding anogenital investigation, the SIM20 group showed a decrease compared with both the CG group (*p* < 0.0001) and the SD group (*p* = 0.0079), whereas the SIM4.2 group showed a decrease only compared with the CG group (*p* = 0.0194) (Figure 4D). For non‐anogenital social interaction, a decrease was observed in the SIM20 group compared with the CG group (*p* = 0.0018) (Figure 4F). In contrast, non‐social exploration increased in both the SIM4.2 and SIM20 groups compared with the untreated groups. Specifically, the SIM20 group showed an increase compared with both the CG group (*p* < 0.0001) and the SD group (*p* = 0.0045), whereas the SIM4.2 group showed an increase compared with the CG group (*p* = 0.0095) (Figure 4E).

**FIGURE 4:**
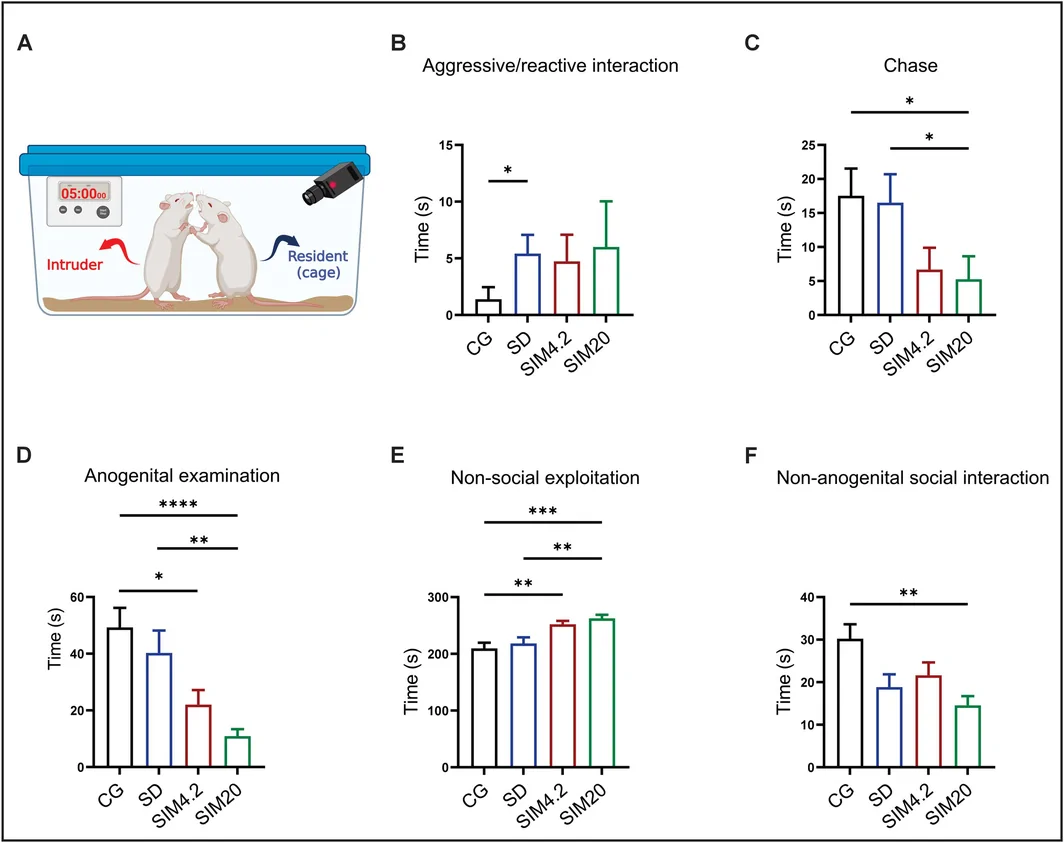
Analysis of the resident‐intruder (RI) test. (A) Schematic diagram of the behavioral paradigm (statistical analysis is not applicable to this panel). (B–F) Graphical representations of the RI test results. Data are expressed as mean ± SEM. Statistical analyses were performed using the Kruskal–Wallis test followed by Dunn's post hoc test (B–E) and one‐way ANOVA followed by Tukey's post hoc test (F). *p < 0.05, **p < 0.01, ***p < 0.001, ****p < 0.0001.

### 3.2 Enzyme‐Linked Immunosorbent Assay Analysis (ELISA)

No statistically significant differences were observed between the groups for the cytokines IL‐4, IL‐6, IL‐10, or TNF‐α (Figure 5A,B,D,F). In contrast, a significant increase in IL‐8 concentration was observed in the SIM20 group (311.7 ± 29.04) compared with the untreated groups (CG, 183.7 ± 3.014; SD, 197.4 ± 6.972), with *p* = 0.0090 and *p* = 0.0142, respectively (Figure 5C). Conversely, TGF‐β concentration was significantly lower in the SIM20 group (234.1 ± 13.34) than in the CG group (324.3 ± 8.354; *p* < 0.0001). A significant reduction was also observed in the SD group (268.7 ± 11.72) compared with the CG group (*p* = 0.0139) (Figure 5E).

**FIGURE 5:**
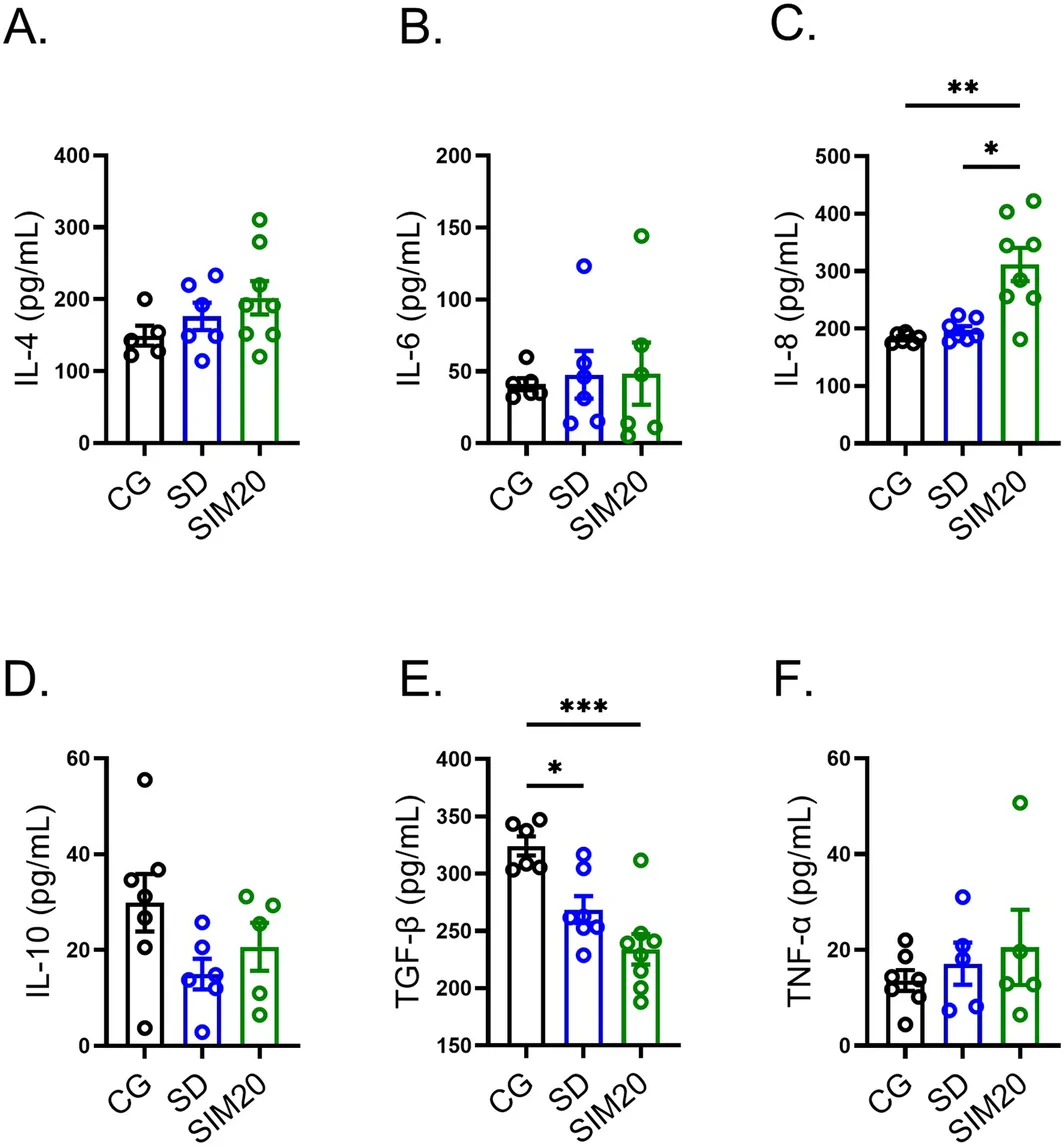
Circulating inflammatory markers. Cytokine concentrations are expressed in pg/mL. (A) IL‐4; (B) IL‐6; (C) IL‐8; (D) IL‐10; (E) TGF‐β; and (F) TNF‐α. Data are expressed as mean ± SEM. Statistical analyses were performed using one‐way ANOVA followed by Tukey's post hoc test (A, D–F) and one‐way Brown‐Forsythe and Welch ANOVA (B–C). CG, *n* = 7; SD, *n* = 7; SIM20, *n* = 8. *p < 0.05, **p < 0.01, ***p < 0.001.

### 3.3 GFAP Immunoreactivity in Dorsal HPC

The GFAP immunohistochemical labeling observed across the four experimental groups and the three major regions of the hippocampal formation (CA1, CA3, and DG) is shown in Figure 6A.

**FIGURE 6:**
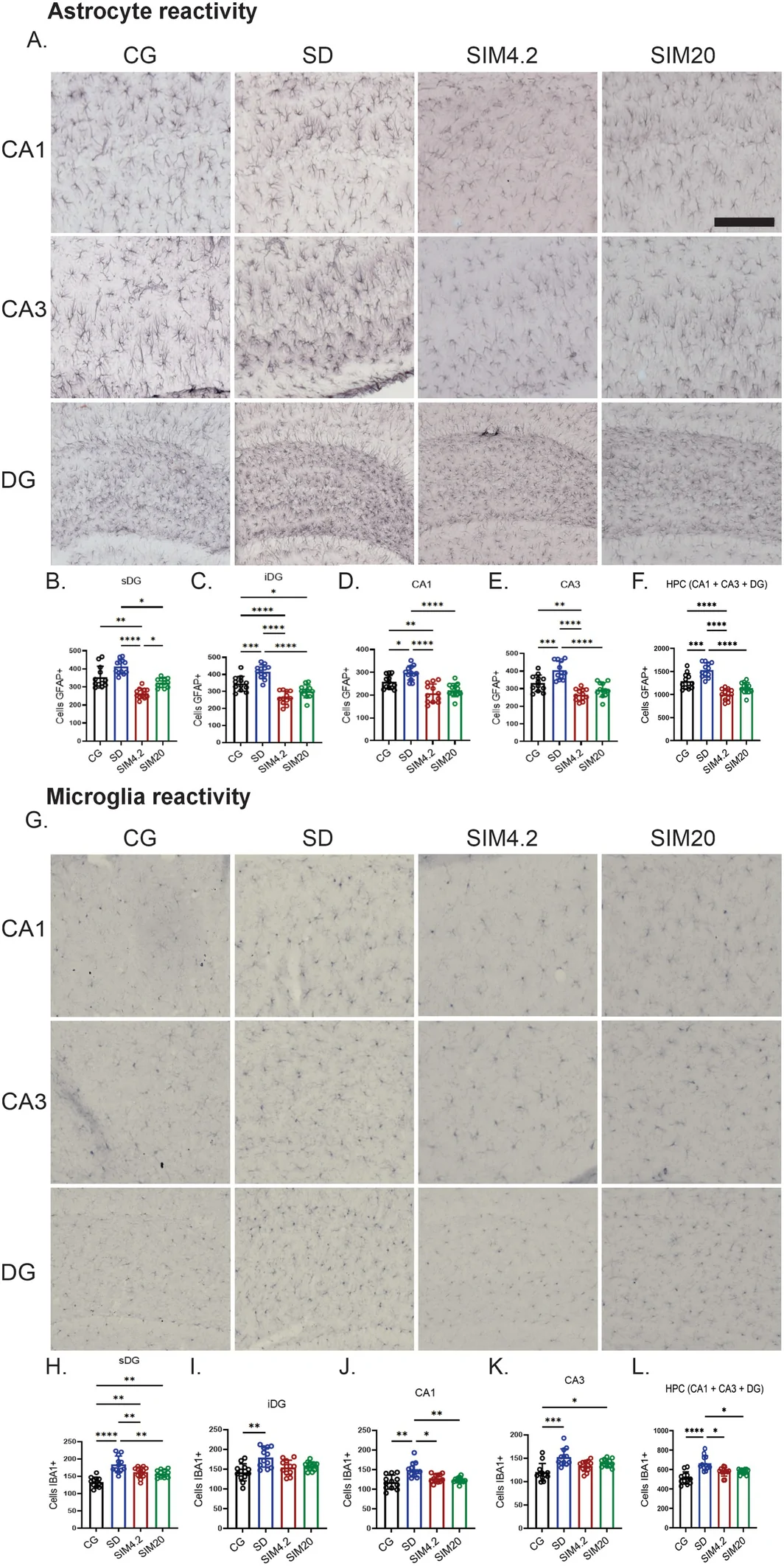
Analysis of GFAP+ and IBA‐1+ immunoreactive cell counts in the dorsal hippocampus (HPC; CA1, CA3, and DG). sDG, superior portion of the dentate gyrus; iDG, inferior portion of the dentate gyrus (*n* = 6 animals/group; right and left HPC analyzed). (A) Representative photomicrographs of GFAP+ immunoreactivity in the CA1, CA3, and DG regions from each experimental group (CG, SD, SIM4.2, and SIM20). (B–F) GFAP+ cell counts in the sDG, iDG, CA1, and CA3, and total dorsal HPC, respectively. (G) Representative photomicrographs of IBA‐1+ immunoreactivity in the CA1, CA3, and DG regions from each experimental group. (H–L) IBA‐1+ cell counts in the sDG, iDG, CA1, and CA3 and total dorsal HPC, respectively. Scale bar = 100 μm. Data are expressed as mean ± SD. Statistical analyses were performed using the Kruskal–Wallis test followed by Dunn's post hoc test (B, K, L), one‐way ANOVA followed by Tukey's post hoc test (C–F, H), and one‐way Brown‐Forsythe and Welch ANOVA (I, J). *p < 0.05, **p < 0.01, ***p < 0.001, ****p < 0.0001.

In the superior portion of the DG, the SD group (411.8 ± 39.71) showed a higher number of astrocytes than the CG group (354.1 ± 59.81; *p* = 0.0057). A reduction in the number of astrocytes was observed in the treated groups (SIM4.2: 263.6 ± 27.21; SIM20: 322.9 ± 24.72) compared with the SD group (*p* < 0.0001). However, the SIM20 group showed a higher number of astrocytes than the SIM4.2 group (*p* = 0.0043) (Figure 6B). In the inferior portion of the DG, the SD group (416.4 ± 40.09) showed a higher number of astrocytes than the CG group (345.6 ± 43.39; *p* = 0.0004). The treated groups showed reduced astrocyte counts (SIM4.2: 264.3 ± 37.71; SIM20: 300.8 ± 37.81) compared with the SD group (*p* < 0.0001). No significant difference was observed between the low‐ and high‐dose treatment groups (Figure 6C).

In the CA1 region, the SD group (297.4 ± 30.98) showed a significant increase in the number of astrocytes compared with the CG group (257.0 ± 29.60; *p* = 0.0214). This increase was attenuated in the treated groups, which showed lower astrocyte counts (SIM4.2: 208.7 ± 40.57; SIM20: 222.1 ± 28.87) compared with the SD group (*p* < 0.0001). No significant difference was observed between the low‐ and high‐dose treatment groups (Figure 6D).

In the CA3 region, a similar pattern was observed, with a higher number of astrocytes in the SD group (404.8 ± 51.17) than in the CG group (331.2 ± 46.48; *p* = 0.0007). This increase was reduced in the treated groups (SIM4.2: 266.2 ± 31.99; SIM20: 294.2 ± 40.02), both of which differed significantly from the SD group (*p* < 0.0001). No significant difference was observed between the two treatment doses (Figure 6E).

For the total dorsal HPC (CA1 + CA3 + sDG + iDG), a higher number of IBA1‐positive cells was observed in the SD group (1530 ± 153) compared with the CG group (1288 ± 165.2; *p* = 0.0007). Simvastatin treatment reduced the number of IBA1‐positive cells (SIM4.2: 1003 ± 118.9; SIM20: 1140 ± 120; *p* < 0.0001). No statistically significant difference was observed between the low‐ and high‐dose treatment groups (Figure 6F).

### 3.4 IBA‐1 Immunoreactivity in Dorsal HPC

The IBA‐1 immunohistochemical labeling observed across the four experimental groups and the three major regions of the hippocampal formation (CA1, CA3, and DG) is shown in Figure 6G.

In the superior portion of the DG, the SD group (184.2 ± 23.83) showed a higher number of IBA‐1‐positive (IBA‐1+) cells than the CG group (133.2 ± 14.06; *p* < 0.0001). A reduction in the number of IBA‐1+ cells was observed in the treated groups (SIM4.2: 161.0 ± 15.58; SIM20: 157.4 ± 10.79) compared with the SD group (*p* = 0.01). No significant difference was observed between the low‐ and high‐dose treatment groups (Figure 6H).

In the inferior portion of the DG, the SD group (179.7 ± 24.69) showed a higher number of IBA‐1+ cells than the CG group (141.8 ± 21.47; *p* = 0.0034). No significant differences were observed in the number of IBA‐1+ cells between the treated groups and the SD group or between the low‐ and high‐dose treatment groups (Figure 6I).

In the CA1 region, the SD group (149.64 ± 18.43) showed a significantly higher number of IBA‐1+ cells than the CG group (118.0 ± 17.65; *p* = 0.0018). This increase was attenuated in both treatment groups (SIM4.2: 127.8 ± 10.14; SIM20: 123.0 ± 7.053) compared with the SD group (*p* = 0.0130 and *p* = 0.0021, respectively). No significant difference was observed between the low‐ and high‐dose treatment groups (Figure 6J).

In the CA3 region, the SD group (152.0 ± 17.99) showed a higher number of IBA‐1+ cells than the CG group (119.8 ± 18.70; *p* = 0.0003). A significant difference was also observed between the CG and SIM20 groups (139.9 ± 8.370; *p* = 0.0374). However, no significant differences were observed between the treated groups and the SD group or between the low‐ and high‐dose treatment groups (Figure 6K).

For the total dorsal HPC (CA1 + CA3 + sDG + iDG), a higher number of GFAP‐positive cells was observed in the SD group (665.5 ± 74.56) compared with the CG group (512.9 ± 62.34; *p* < 0.0001). Simvastatin treatment reduced the number of GFAP‐positive cells (SIM4.2: 576.6 ± 44.52; SIM20: 577.9 ± 24.51; *p* = 0.05). No statistically significant difference was observed between the low‐ and high‐dose treatment groups (Figure 6L).

### 3.5 GFAP and IBA‐1 Immunoreactivity in the Amygdala

GFAP immunoreactivity across the four experimental groups in the BLA and CeA of the AMY is shown in Figure 7A. In the BLA, no statistically significant differences in GFAP immunoreactivity were observed among the experimental groups (Figure 7B). In the CeA, the SIM20 group (129.2 ± 10.75) showed a higher number of GFAP‐positive cells than the SIM4.2 group (82.63 ± 16.89; *p* = 0.0025) (Figure 7C).

**FIGURE 7:**
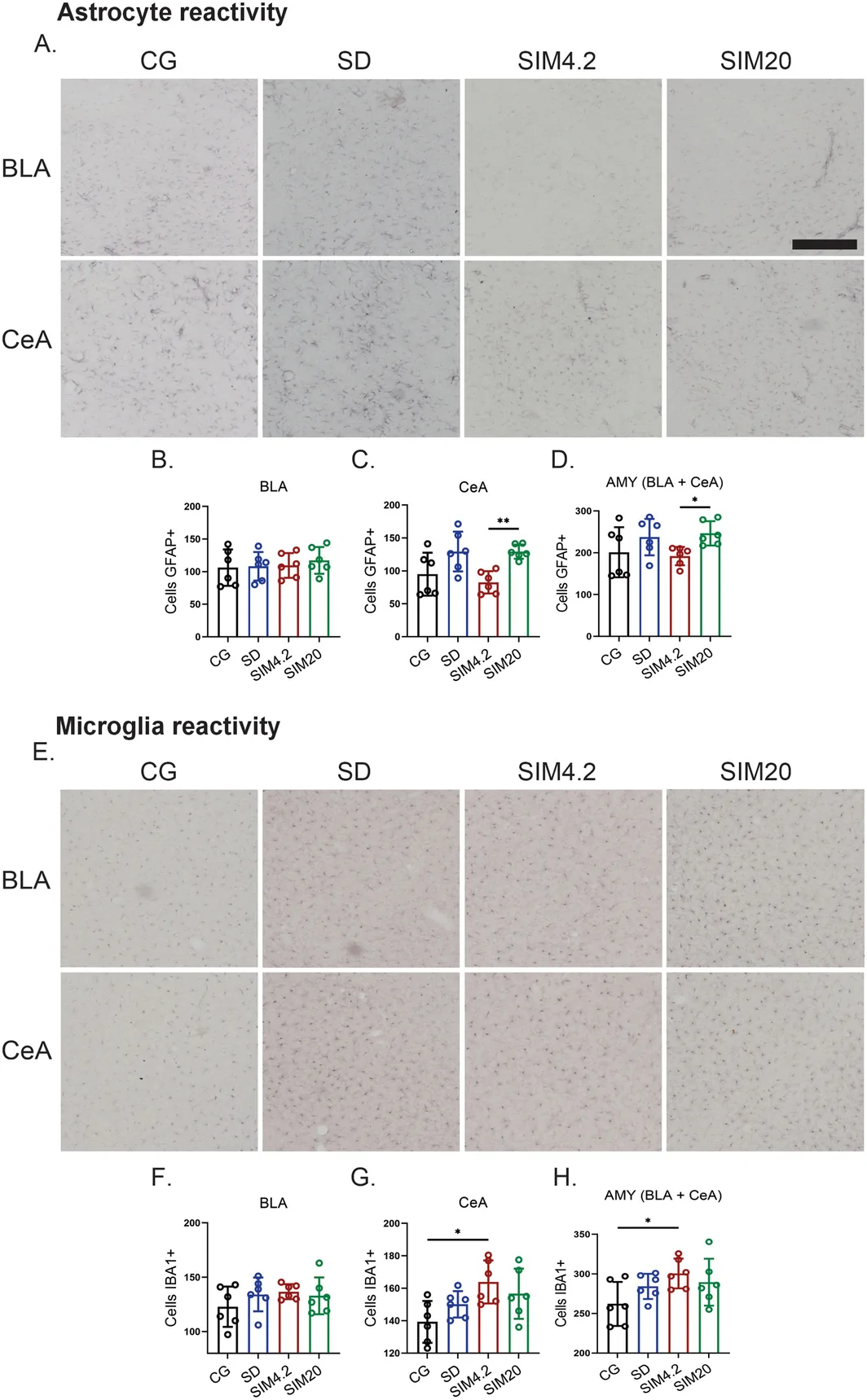
Analysis of GFAP+ and IBA‐1+ immunoreactive cell counts in the amygdala (AMY; BLA and CeA). Data are expressed as mean ± SD (*n* = 6 animals/group; right and left AMY were analyzed). (A) Representative photomicrographs of GFAP+ immunoreactivity in the BLA and CeA regions from each experimental group (CG, SD, SIM4.2, and SIM20). (B–D) GFAP+ cell counts in the BLA, CeA, and total AMY, respectively. (E) Representative photomicrographs of IBA‐1+ immunoreactivity in the BLA and CeA regions from each experimental group. (F–H) IBA‐1+ cell counts in the BLA, CeA, and total AMY, respectively. Scale bar = 100 μm. Statistical analyses were performed using one‐way ANOVA followed by Tukey's post hoc test (B, F–H) and one‐way Brown‐Forsythe and Welch ANOVA (C, D). *p < 0.05, **p < 0.01.

For GFAP‐positive cells in the total amygdala (BLA + CeA), an increase was observed in the SIM20 group (246.4 ± 29.15) compared with the SIM4.2 group (192.1 ± 22.05; *p* = 0.0286). No statistically significant difference was observed between the SD and CG groups (Figure 7D).

IBA‐1 immunoreactivity across experimental groups in the basolateral (BLA) and central (CeA) nuclei of the amygdala (AMY) is presented in Figure 7E–H. In the BLA, no statistically significant differences in the number of IBA‐1‐positive cells were observed among groups (Figure 7F). In contrast, analysis of the CeA revealed a significantly higher number of IBA‐1‐positive cells in the SIM4.2 group (163.9 ± 13.30) compared to the CG group (139.3 ± 12.89; *p* = 0.0155; Figure 7G). When evaluating the total amygdala (BLA + CeA combined), the SIM4.2 group also exhibited a significantly higher count (300.6 ± 18.77) than the CG group (262.1 ± 27.77; *p* = 0.0492; Figure 7H). No statistically significant differences were detected between the SD and CG groups (Figure 7F–H).

### 3.6 Microglial Morphological Profiling and Region‐Specific Phenotypic Redistribution

The three microglial morphological profiles were clearly identified: Type I (surveillance/resting), Type II (transitional), and Type III (hypertrophic/reactive). The morphological features of these three microglial categories are shown in Figure 8A.

**FIGURE 8:**
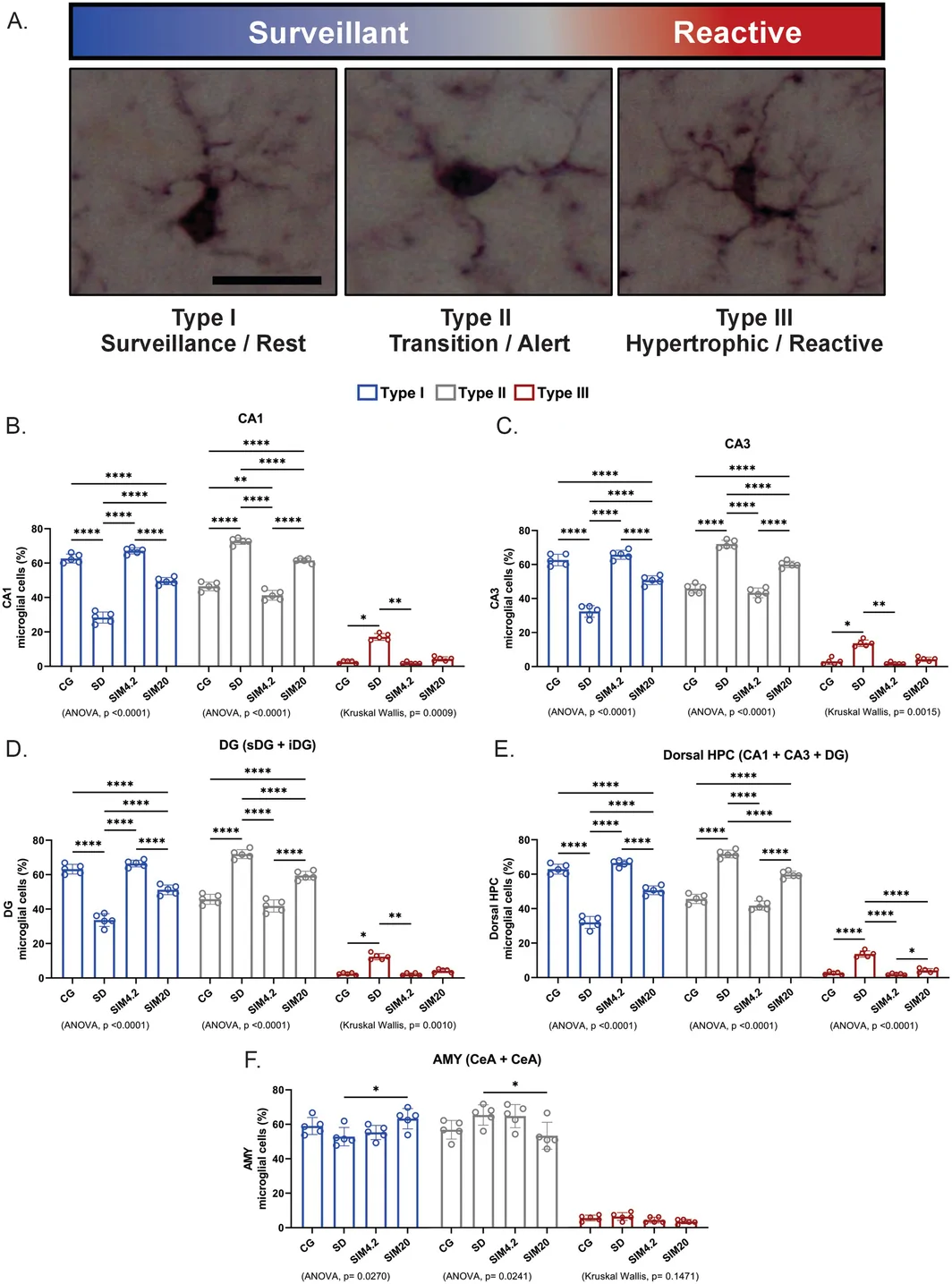
Microglial morphological shifts and phenotypic distribution across hippocampal subregions and amygdaloid nuclei following sleep deprivation and simvastatin treatment. (A) Representative micrographs illustrating the morphological continuum of microglia categorized into three distinct phenotypes: Type I (Surveillance/Rest), Type II (Transition/Alert), and Type III (Hypertrophic/Reactive). Scale bar = 50 μm. (B–F) Quantitative analysis of microglial phenotypic distribution (percentage of cells) in the hippocampal regions (CA1, CA3, and total DG; B–E) and the total amygdala (AMY; F) across the experimental groups: Control (CG), Sleep Deprivation (SD), Sleep Deprivation + Low‐Dose Simvastatin (SIM4.2), and Sleep Deprivation + High‐Dose Simvastatin (SIM20). Bar graphs show the percentage of cells corresponding to each morphological phenotype. Data are expressed as mean ± SD. Statistical analysis was performed using one‐way ANOVA followed by Tukey's multiple‐comparisons test. *p ≤ 0.05, **p ≤ 0.01, ****p ≤ 0.0001.

In the dorsal HPC, sleep deprivation induced a marked shift in microglial morphology, characterized by a reduction in Type I microglia and increases in Type II and Type III phenotypes compared with the CG group (all *p* < 0.0001) (Figure 8B–E). Both SIM4.2 and SIM20 significantly shifted the microglial profile toward the homeostatic phenotype, with increased proportions of Type I and reduced proportions of Type II and Type III microglia compared with the SD group (all *p* < 0.0001) (Figure 8B–E). However, SIM4.2 restored the microglial profile to levels comparable to those observed in the CG group, whereas SIM20 maintained an intermediate phenotype.

In the AMY, no major changes in microglial phenotype were detected. The only significant differences were a modest increase in Type I microglia in the SIM20 group compared with the SD group (*p* = 0.0243) and a corresponding decrease in Type II microglia (*p* = 0.0457) (Figure 8F).

### 3.7 Correlation Analysis Between Behavioral and Glial Parameters

Correlation analyses revealed significant associations among several behavioral parameters assessed in the resident‐intruder (RI) test (Figure 4). Aggressive/reactive interaction showed a positive correlation with chasing behavior (*r* = 0.73, *p* = 0.010) and a strong negative correlation with non‐social exploration (*r* = −0.73, *p* = 0.010). Non‐social exploration was negatively correlated with both anogenital investigation (*r* = −0.90, *p* < 0.001) and chasing behavior (*r* = −0.87, *p* < 0.001). In contrast, anogenital investigation showed a strong positive correlation with chasing behavior (*r* = 0.83, *p* = 0.002). No significant correlations were observed between IBA‐1+ or GFAP+ cell counts in the total dorsal HPC or AMY and any of the behavioral parameters assessed (Figure 9).

**FIGURE 9:**
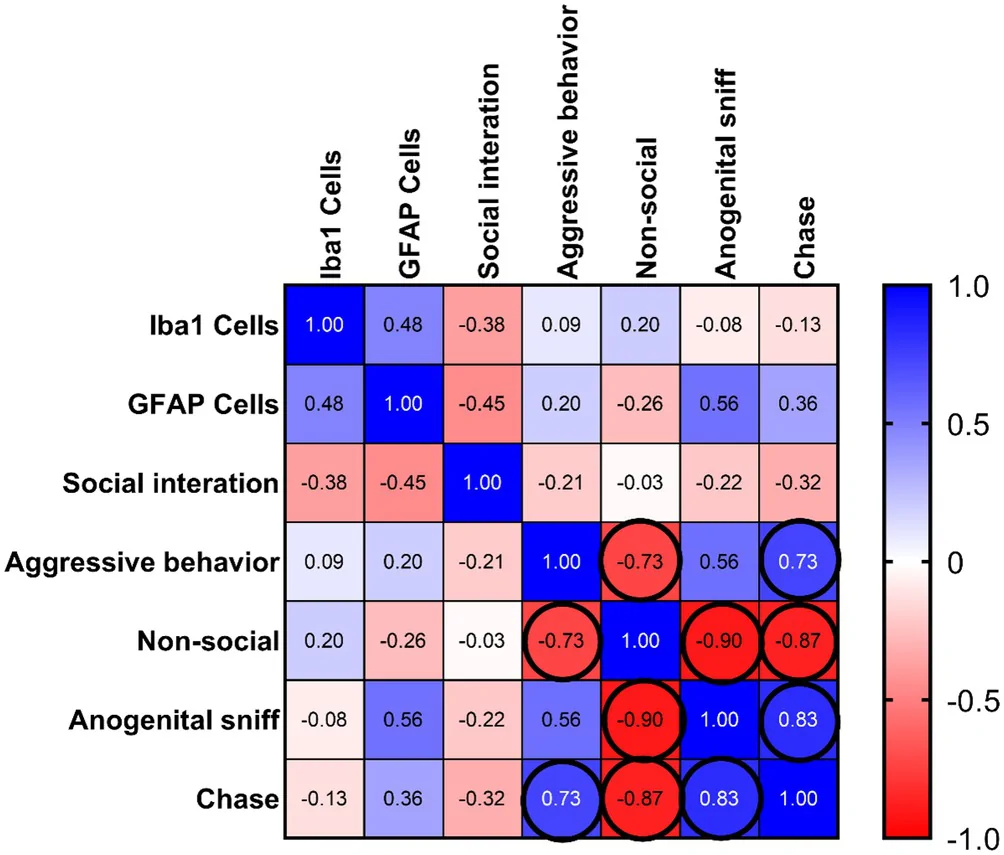
Heatmap showing the Pearson correlation coefficient matrix between glial cell counts and behavioral variables. Black circles indicate statistically significant correlations (*p* < 0.05). Cell counts from the total dorsal hippocampus (HPC) were used for the correlation analysis. Results are presented as Pearson correlation coefficients (*r*).

## 4 Discussion

To our knowledge, no previous studies have directly investigated the association between statin treatment and reduced astrocytic and microglial immunoreactivity in the context of SD. Therefore, our findings provide novel evidence that statin treatment may attenuate glial activation under SD conditions, potentially modulating its effects on central nervous system structure and function.

This study demonstrates that SD acts as a potent biological stressor, inducing behavioral disturbances and region‐specific glial remodeling within the limbic system. SD promoted aggressive behavior accompanied by increased astrocytic and microglial reactivity in the dorsal HPC, along with a significant shift from a homeostatic (Type I) toward reactive (Types II and III) microglial phenotype. Daily simvastatin treatment significantly attenuated these alterations, restoring a more homeostatic glial profile in the dorsal HPC while also reducing aggressive behavior and increasing exploratory activity. In contrast, glial alterations in the AMY were comparatively subtle, highlighting the regional heterogeneity of the neuroinflammatory response to SD. Analysis of peripheral cytokines further revealed selective modulation of systemic inflammatory mediators following simvastatin treatment. Taken together, these findings suggest that simvastatin may modulate both the behavioral and neuroinflammatory consequences of chronic SD.

The RI test showed reduced social interaction following simvastatin treatment, particularly in the SIM20 group, consistent with previous reports of reduced social investigative behavior following simvastatin administration (Segatto et al. 2014). Although SD increased aggressive interactions compared with the CG, this effect was not attenuated by simvastatin treatment. The magnitude of aggression was lower than that reported in more aggressive strains, such as wild‐type Groningen rats, which may reflect the naturally lower baseline levels of aggression in Wistar rats (Koolhaas et al. 2013).

Chasing and anogenital investigation, behaviors associated with social recognition and hierarchical interactions (Blanchard and Blanchard 1977; Wesson 2013), were significantly reduced following simvastatin treatment, particularly in the SIM20 group. Together with the increase in non‐social exploration, these findings suggest a shift in behavioral allocation away from social interaction and toward environmental exploration, rather than a generalized suppression of aggressive behavior by simvastatin. This interpretation is consistent with previous studies reporting antidepressant‐ and anxiolytic‐like effects of simvastatin, characterized by increased exploratory and active behaviors in rodents exposed to stressful conditions (Lin et al. 2014; Naserzadeh et al. 2019).

Peripheral cytokine analysis revealed that SD and simvastatin treatment were associated with selective rather than generalized changes in systemic inflammatory mediators. Rather than producing uniform changes across the cytokines evaluated, simvastatin was associated with modulation of specific inflammatory mediators, consistent with the pleiotropic immunomodulatory effects attributed to statins beyond their cholesterol‐lowering properties and related to inhibition of the mevalonate pathway (S. W. Kim et al. 2019). Although previous studies have reported increased inflammatory cytokine levels following sleep deprivation (Gunawan et al. 2025; Hurtado‐Alvarado et al. 2016; N. U. K. Niazi et al. 2025; Zielinski et al. 2014), SD alone did not significantly alter the systemic cytokine profile in the present study. These findings indicate that systemic inflammatory responses to prolonged sleep deprivation may be context‐dependent and influenced by differences in the experimental model and methodological design.

Among the cytokines evaluated, TGF‐β was significantly reduced in the SIM20 group. In addition to its well‐established roles in tissue repair and remodeling, TGF‐β regulates astrocyte proliferation and extracellular matrix remodeling (Lindholm et al. 1992). Simvastatin has been shown to attenuate TGF‐β‐dependent signaling through inhibition of the mevalonate pathway (M.‐L. Kim et al. 2017), providing a potential explanation for the reduction observed in the present study. Nevertheless, circulating TGF‐β concentrations do not necessarily reflect TGF‐β signaling within the brain parenchyma, where this pathway plays an important role in maintaining microglial homeostasis and identity (Krzyzan et al. 2026).

TNF‐α and IL‐6 remained unchanged after 45 days of SD, consistent with evidence that these cytokines are predominantly elevated during the early phases of sleep deprivation (Irwin et al. 2006). In contrast, IL‐8 was selectively increased in the SIM20 group. Although classically regarded as a pro‐inflammatory chemokine, IL‐8 also participates in endothelial repair, and simvastatin has been reported to enhance its expression in vascular cells (Park et al. 2008). Given that SD can impair endothelial function (Holmer et al. 2021), the increase in IL‐8 observed in the SIM20 group may reflect a compensatory response related to vascular homeostasis rather than an exacerbation of systemic inflammation. Although cytokines were measured only in the circulation, these findings suggest that selective peripheral immune modulation may accompany the attenuation of glial reactivity observed in the dorsal HPC.

Taken together, the peripheral cytokine findings suggest that simvastatin did not induce a generalized anti‐inflammatory response after prolonged sleep deprivation, but rather produced a selective modulation of systemic immune mediators. The increase in IL‐8 in the SIM20 group, together with the reduction in TGF‐β, indicates that simvastatin altered specific components of the circulating inflammatory profile without broadly suppressing cytokine production. The increase in IL‐8 may represent a compensatory response related to endothelial homeostasis, whereas the reduction in circulating TGF‐β may reflect modulation of the mevalonate pathway and TGF‐β‐dependent signaling. However, because these cytokines were measured only in the circulation, their relationship with regional changes in astrocytic and microglial reactivity cannot be established directly. Nevertheless, the coexistence of selective peripheral cytokine changes with reduced GFAP and IBA‐1 immunoreactivity and a shift toward a more homeostatic microglial phenotype in the dorsal HPC suggests that simvastatin may exert coordinated but compartment‐specific immunomodulatory effects during prolonged sleep deprivation. Importantly, these effects were not restricted to a single glial population, as simvastatin was associated with changes in both astrocytic and microglial responses, suggesting a broader modulation of glial reactivity rather than a cell‐type‐specific effect.

GFAP immunoreactivity in the dorsal HPC revealed a significant increase in astrocyte counts in the SD group across all analyzed subregions (CA1, CA3, sDG, and iDG) compared with the CG group. Simvastatin treatment reduced astrocyte counts in all subregions, suggesting a modulatory effect on astrocytic reactivity. Increased GFAP immunoreactivity has previously been reported in both acute and chronic sleep deprivation models; however, findings across studies have been inconsistent. For example, increased GFAP immunoreactivity was observed in the hippocampus after 12 h of acute sleep deprivation (Mishra et al. 2016), whereas no significant increase was detected after 24, 48, or 72 h in the CA1, CA3, or DG regions of the rat hippocampus (Wadhwa, Kumari, et al. 2017). Similarly, a 12‐h acute sleep deprivation study reported increased IBA‐1 levels in the striatum but no significant change in GFAP immunoreactivity in the hippocampus (Avilez‐Avilez et al. 2025). These discrepancies suggest that the astrocytic response to sleep deprivation may depend on the duration and experimental characteristics of the sleep deprivation protocol, with prolonged exposure potentially being required to elicit more robust GFAP alterations (Avilez‐Avilez et al. 2025; Hou et al. 2019; Hsu et al. 2003). The reduction in GFAP immunoreactivity observed following simvastatin treatment is consistent with previous evidence that simvastatin can attenuate astrocytic and microglial activation in models of traumatic brain injury, suggesting potential neuroprotective effects associated with modulation of glial reactivity (Li et al. 2009).

IBA‐1 immunoreactivity in the dorsal HPC revealed an increase in microglial cell counts in the SD group across all analyzed subregions compared with the CG group, consistent with the pattern observed for GFAP. These findings are in agreement with previous studies reporting increased microglial reactivity following sleep deprivation (Bellesi et al. 2017; Wadhwa, Prabhakar, et al. 2017). Although the glymphatic system was not directly evaluated in the present study, microglia interact closely with astrocytic processes expressing aquaporin‐4 (AQP4), a key component of the glymphatic clearance system (Bellesi et al. 2017; Haruwaka et al. 2019). In this context, previous evidence has shown that sleep deprivation can impair glymphatic function and cognitive performance (S. Deng, Hu, et al. 2024), providing a potential framework for interpreting the concurrent astrocytic and microglial alterations observed in the present study.

Microglial morphology across the hippocampal subregions (CA1, CA3, iDG, and sDG) and the basolateral amygdala (BLA) revealed region‐specific susceptibility to SD, with a shift in the microglial population from a surveillance phenotype (Type I) toward intermediate (Type II) and hypertrophic/reactive (Type III) phenotypes. Rather than remaining quiescent, surveillance microglia continuously monitor the parenchymal environment and undergo dynamic morphological adaptations in response to local microenvironmental changes (Illes et al. 2021; J. Kim et al. 2024; Xu et al. 2022). Accordingly, SD was associated with a marked accumulation of transitional Type II cells, whereas a smaller proportion exhibited the hypertrophic Type III phenotype, consistent with the low‐grade neuroinflammatory profile described in sleep restriction models (Manchanda et al. 2018; N. U. K. Niazi et al. 2024; Woodburn et al. 2021). Together, these findings suggest that chronic sleep loss disrupts local microglial homeostasis and induces moderate, region‐specific glial remodeling without evidence of overt neuroinflammation (Bellesi et al. 2017; Liu et al. 2022; Parhizkar et al. 2023).

Both doses of simvastatin markedly reduced the proportion of Type III hypertrophic microglia, whereas the higher dose maintained a predominance of transitional Type II cells, supporting a modulatory effect on microglial morphological reactivity. This pattern is consistent with current models describing microglial activation as a continuous phenotypic spectrum rather than a binary process (Biswas 2023; Illes et al. 2021; Lier et al. 2021; Valiukas et al. 2025). Nevertheless, morphological changes alone cannot define microglial effector function (J. Kim et al. 2024; Woodburn et al. 2021). Therefore, although simvastatin attenuated hypertrophic morphological reactivity and shifted the microglial profile toward transitional phenotypes, additional transcriptional and functional analyses are required to determine whether these morphological changes correspond to a distinct anti‐inflammatory or pro‐homeostatic state.

Unlike the dorsal HPC, astrocytic and microglial responses in the AMY were comparatively limited, further supporting the concept of regional heterogeneity in glial susceptibility to SD. No significant differences in GFAP‐ or IBA‐1‐positive cell counts were detected in the BLA, consistent with previous reports suggesting that stress‐related glial responses may be more pronounced in the hippocampus than in the BLA (Naskar and Chattarji 2019). In contrast, subtle dose‐dependent differences were observed in the CeA, with higher GFAP immunoreactivity in the SIM20 group and higher IBA‐1 immunoreactivity in the SIM4.2 group. These findings suggest that individual amygdaloid nuclei may exhibit distinct responses to prolonged sleep deprivation and simvastatin treatment.

Recent evidence indicates that CeA astrocytes actively participate in the regulation of emotional behaviors through bidirectional communication with local neuronal circuits (Zan et al. 2025). Although astrocytic function was not directly assessed in the present study, the modest glial alterations observed in the CeA may reflect nucleus‐specific adaptations rather than robust neuroinflammatory activation. Similarly, the limited microglial changes observed in the AMY further support the notion that SD may affect glial populations in the hippocampus and amygdala differently, highlighting the regional specificity of glial responses within the limbic system (Wohleb et al. 2013).

The present study demonstrated that SD induced a pronounced shift from homeostatic (Type I) toward reactive microglial phenotypes in the HPC. These findings are consistent with recent evidence showing that sleep deprivation induces region‐dependent remodeling of hippocampal microglia, affecting cell morphology, density, and phagocytic activity depending on both the hippocampal subregion and the sleep deprivation paradigm employed (Kox et al. 2026). However, whereas Kox et al. (2026) investigated acute sleep deprivation, our study extends these observations to a chronic sleep deprivation model and provides evidence that simvastatin attenuates these morphological alterations.

The reduction in IBA‐1‐positive cell counts observed in the simvastatin‐treated groups may be associated with modulation of the NF‐κB signaling pathway, which plays an important role in microglial activation and inflammatory cytokine production. Previous studies have shown that simvastatin can inhibit NF‐κB signaling and attenuate microglial inflammatory responses (Cordle and Landreth 2005; Lee et al. 2007; Lietzau et al. 2023). Thus, modulation of this pathway represents a potential mechanism underlying the attenuation of microglial reactivity observed following simvastatin treatment, although this mechanism was not directly assessed in the present study.

Comparisons between the SIM4.2 and SIM20 groups revealed no significant differences in most glial parameters, indicating that both doses produced a comparable attenuation of sleep deprivation‐induced glial reactivity. These findings suggest the absence of a clear dose‐dependent effect within the dose range evaluated and may indicate that the lower dose was sufficient to elicit substantial glial modulation. This pattern may be related, at least in part, to the high lipophilicity and blood–brain barrier (BBB) permeability of simvastatin (Guo et al. 2021). Although the underlying mechanisms were not directly investigated, inhibition of HMG‐CoA reductase reduces the synthesis of isoprenoid intermediates required for the prenylation of small GTPases involved in glial signaling, representing one of the principal pleiotropic mechanisms proposed for statins in the central nervous system (Alizadeh et al. 2017; Bi et al. 2004). In contrast, some behavioral parameters exhibited a different response profile, with SIM20 reaching statistical significance relative to both CG and SD, whereas SIM4.2 did not. Together, these findings indicate that the effects of simvastatin were not uniformly dose‐dependent across behavioral and glial outcomes. Notably, there was no evidence of reduced efficacy at the higher dose. Although paradoxical effects of simvastatin have been reported, these effects were observed at substantially higher, supratherapeutic doses (e.g., 88 mg/kg) than those used in the present study (Okudan and Belviranli 2020), suggesting that the doses used here remained within a range capable of producing biological effects without evidence of an apparent loss of efficacy at the higher dose. Thus, the absence of a dose‐dependent effect on glial parameters, together with the greater behavioral sensitivity to the higher dose, suggests that behavioral and glial responses to simvastatin may involve partially distinct dose–response relationships.

The concomitant attenuation of GFAP and IBA‐1 immunoreactivity suggests that simvastatin may modulate glial crosstalk. Although this interaction was not directly investigated in the present study, previous evidence indicates that microglia can influence astrocytic activation through NF‐κB‐dependent inflammatory signaling (Liddelow et al. 2017; Matejuk and Ransohoff 2020). Thus, the coordinated changes observed in astrocytic and microglial markers may reflect, at least in part, modulation of intercellular signaling between these glial populations.

The present findings indicate that SD was associated with both behavioral alterations and glial changes, whereas simvastatin modulated both outcomes. However, no significant correlations were observed between glial parameters and behavioral measures in the dorsal HPC. Thus, the present study does not provide evidence for a direct association between the glial alterations and behavioral changes, nor does it establish a causal relationship between these outcomes. Future studies employing pharmacological or genetic approaches to selectively manipulate astrocytes and microglia will be necessary to determine whether these cellular alterations contribute causally to the behavioral changes associated with SD or instead represent parallel responses to sleep deprivation (Lehmann et al. 2019; Prinz et al. 2019).

This study has several limitations that should be considered. First, only male rats were included in the experimental design. Although the use of male animals was intended to improve experimental standardization and was based on evidence that the resident‐intruder (RI) paradigm generally produces more robust and consistent behavioral responses in males, the exclusive use of male animals limits the generalizability of the findings (Koolhaas et al. 2013). Recent studies suggest that sex‐specific biological factors may influence sleep regulation, inflammatory and glial responses, and cognitive and emotional outcomes following sleep deprivation (Hajali et al. 2019; Wright et al. 2023). Furthermore, sex‐dependent differences in glial biology have increasingly been recognized. Therefore, future studies including female animals are needed to determine whether the effects of chronic sleep deprivation and simvastatin observed in the present study are consistent across sexes.

Although GFAP and IBA‐1 provide important markers of astrocytic and microglial reactivity, respectively, they do not fully capture the phenotypic and functional heterogeneity of these cell populations. Further studies incorporating more comprehensive morphological, molecular, and functional approaches are needed to elucidate the cellular mechanisms linking chronic sleep deprivation, simvastatin‐mediated glial modulation, and behavioral outcomes.

Another limitation of this study is the absence of molecular analyses investigating the signaling pathways underlying the observed glial alterations. Although changes in astrocytic and microglial immunoreactivity and morphology provide evidence of glial remodeling following chronic sleep deprivation and simvastatin treatment, these approaches do not determine the molecular mechanisms underlying these effects. Future studies incorporating gene and protein expression analyses, together with assessment of key neuroinflammatory signaling pathways, including NF‐κB and the NLRP3 inflammasome, will be important to clarify the molecular mechanisms underlying the glial responses described here.

Additionally, circulating cytokine concentrations may not accurately reflect inflammatory signaling within the brain, limiting the extent to which peripheral immune responses can be directly linked to central glial alterations.

The present findings demonstrate that chronic sleep deprivation is associated with region‐specific glial responses, characterized by marked astrocytic and microglial alterations in the dorsal HPC but comparatively limited changes in the AMY. Simvastatin attenuated several of these glial alterations and shifted the microglial profile toward a more homeostatic phenotype, supporting its potential to modulate sleep deprivation‐induced glial remodeling. These observations reinforce the concept that glial responses to chronic stressors are not uniform throughout the brain but are influenced by the local microenvironment and the specific properties of each neural circuit. Furthermore, the magnitude and pattern of these responses may vary according to the sleep deprivation paradigm employed and its duration.

Simvastatin modulated these glial responses in a region‐specific and not uniformly dose‐dependent manner, attenuating glial alterations in the dorsal HPC while producing more subtle and distinct effects in the AMY. These effects may involve mechanisms beyond HMG‐CoA reductase inhibition, including reduced isoprenoid‐dependent signaling and attenuation of NF‐κB‐mediated inflammatory pathways, as well as potential preservation of neurovascular and blood–brain barrier homeostasis. However, these mechanisms were not directly assessed in the present study and should therefore be considered potential explanations rather than established mechanisms. Taken together, these findings support the pleiotropic and context‐sensitive nature of statin action and suggest that its effects on the central nervous system extend beyond classical anti‐inflammatory mechanisms to include modulation of glial homeostasis.

## Author Contributions

Higor Hernany Dias: conceptualization, formal analysis, investigation, methodology, writing – original draft, writing – review and editing. Kevin Silva Muller: methodology, formal analysis, writing – review and editing. Gabriela Larissa Lima‐da‐Silva: conceptualization, investigation, methodology, writing – review and editing. Stephane Silva Alves: methodology, writing – review and editing. Tatiane de Camargo Martins: methodology, writing – review and editing. Pedro Tadao Hamamoto Filho: funding acquisition, methodology, writing – review and editing; José de Anchieta de Castro e Horta‐Júnior: conceptualization, funding acquisition, data curation, formal analysis, investigation, methodology, writing – original draft, writing – review and editing.

## Funding

This study was funded by the Conselho Nacional de Desenvolvimento Científico e Tecnológico (Brazil) which provided a scholarship to Stephane da Silva Alves (130808/2023‐7), Higor Hernany Dias (140201/2026‐2) and Pedro Tadao Hamamoto Filho (313047/2023‐5), the Coordenação de Aperfeiçoamento de Pessoal de Nível Superior (CAPES), Brazil—Finance Code 001, which provided a scholarship to Higor Hernany Dias (88887.948051/2024‐00) and Stephane da Silva Alves (88887.915334/2023‐00), and by the São Paulo Research Foundation (FAPESP), Brazil, which provided grant support to José de Anchieta de Castro e Horta‐Júnior (2016/13136‐6) and Pedro Tadao Hamamoto Filho (22/03042‐5), and a scholarship to Gabriela Larissa Lima da Silva (2022/16417‐7), Kevin Silva Müller (2022/0755‐1), and Tatiane de Camargo Martins (23/12860‐6). The funding sources had no involvement in the study design, data collection and analysis, interpretation of the results, preparation of the manuscript, or the decision to submit it for publication.

## Declaration of Transparency

The authors, reviewers and editors affirm that in accordance to the policies set by the *Journal of Neuroscience Research*, this manuscript presents an accurate and transparent account of the study being reported and that all critical details describing the methods and results are present.

## Conflicts of Interest

The authors declare no conflicts of interest.

## Supporting information


**Data S1:** Photos of modified multi‐platform (MMP), with 18 acrylic platforms across the water tank used to induce sleep deprivation.
**Data S2:** Photo of doses of edible small pellets formed by a mixture of simvastatin and a powdered base for preparing whipped cream, used for the administration of the vehicle and the statins.

**Appendix S1:** Transparent Science Questionnaire for Authors.

## Data Availability

The data that support the findings of this study are available from the corresponding author upon reasonable request.
