# Supplementary material for: Simvastatin Modulates Astrocyte and Microglial Reactivity and Aggressive Behavior After Chronic Sleep Deprivation in Rats
Source: J Neurosci Res. 2026 Nov 1;104(11):e70160. doi: 10.1002/jnr.70160 (PMC13724290; doi:10.1002/jnr.70160)
Supplement: Supplementary file 1 [file JNR-104-e70160-s002.pdf]

JOURNAL OF Neuroscience Research

Rigorous study design and transparent reporting of results are the cornerstones of science. By maximizing the information provided in a manuscript, factors that may contribute to irreproducibility will be mitigated. The *Journal of Neuroscience Research* promotes transparency in research by strongly encouraging authors to include all relevant information about their studies (see our preprint for details). To expedite reviewer monitoring of these factors, authors submitting original research articles must complete this questionnaire.

If the manuscript is accepted and all items within the checklist are present, we will include a declaration of transparency at the end of the manuscript. This declaration reads as follows:

> ***The authors, reviewers and editors affirm that in accordance to the policies set by the Journal of Neuroscience Research, this manuscript presents an accurate and transparent account of the study being reported and that all critical details describing the methods and results are present***.

To complete the checklist, fill in the right-hand column with the page and paragraph number (e.g., 'Page 3, Paragraph 2') corresponding to the checklist item. If a checklist item is not applicable to the study being reported or the authors are unable to provide that item, a reason must be supplied. Additional comments can be added at the end of the document. Upload the completed document as supplementary information for review.

Experimental and Study Design	
1. Clearly state the primary and any secondary objective or hypothesis of the study	Sleep deprivation causes changes in glial reactivity, social behavior, and aggression, and simvastatin reduces these changes.
2. For each experiment, the study design must include:<br>*a. Number of experimental and control groups*	3 groups and 1 control group
b. Randomization and blinding procedures and/or steps to minimize subjective bias when allocating subjects to experimental groups	the experimental groups were randomly divided
c. Precise details of all procedures, including housing and husbandry are carried out in the experiment<br>*d. Is sex considered as a biological variable? See Editorial for details about proper reporting*	All procedures were approved by the Institutional Animal Care and Use Committee of São Paulo State University (Unesp) – Botucatu, SP, Brazil (CEUA-1414/2022) and conducted in accordance with ethical guidelines for animal experimentation (CONCEA). Male Wistar rats (Rattus norvegicus) were obtained from the University of São Paulo breeding facility and housed under controlled conditions of temperature (22 ± 2 °C); and luminosity (12 h light/dark cycle, lights on at 5:00 p.m. until

JOURNAL OF Neuroscience Research

	5:00 a.m.), filtered water and Presence® feed ad libitum. Animals were maintained in pairs or trios in standard cages with environmental enrichment. The rats were kept in the animal facility of the Toxicological Assistance Center (CEATOX) of the Institute of Biosciences (IB) at Unesp in Botucatu until they reached 75 days of age.
	Yes, they are variable, mainly due to the behavior that is better in male rats.

Experimental Subjects

3. Specify the total number of subjects in each experiment, including the number of animals, sex and age in each group<br>*a. Explain how the number of animals were arrived at and provide details of any sample size calculation, including power analysis*<br>*b. Indicate the number of independent replications of each experiment, when applicable*	6 animals for morphological analysis and 15 animals for behavior test. Male rats with 75 days of age.
	No replies

Data Handling

4. Indicate data collection start and stop rules:<br>*a. Define the criteria for data/subject inclusion and exclusion. If any outcome or condition measure used was not reported in the results section, authors must address this omission*	No data was excluded from the study
b. Specify reasons for any discrepancy between the number of animals at the beginning and end of the study	No discrepancies between the number of animals
c. Define and explain how outliers are handled and report if data are removed prior to analysis	Outlier tests with rout=2%

Statistical Analysis and Depiction of Continuous Data

5. Provide details of the statistical methods used for each analysis<br>*a. State, define and justify the statistical analysis used and specify the unit of analysis for each dataset*<br>*b. Describe and report methods used to assess whether data met the assumptions of the statistical approach and any adjustments for multiple comparisons*<br>*c. Fully report statistics (including exact value of N, degrees of freedom, test value and exact P-value when >0.001) and we encourage the use of effect sizes and confidence intervals*<br>*d. Disaggregated data are presented for males and females*	We used one-way ANOVA for parametric data and Kruskal-Wallis for nonparametric data (behavioral and morphological tests).
	Normality tests (Shapiro-Wilk) and verification of variances between data

e. Data distribution is depicted with univariate scatterplots boxplots, violin plots, or kernel density plots when presenting ***continuous data*** *(see Editorial* [Publishing Transparent and Rigorous Scientific Research](#)*)*	We mainly used mean and standard error of the mean (SEM) for behavioral data and mean and standard deviation (SD) for morphological data, followed by the exact P test

Discussion

6. Comment on study limitations including any potential source of bias, limitations to the animal model, imprecisions associated with the results, and the inability for any reason to study possible sex influences where they may exist.	Only GFAP and IBA1 markers were used. The intruder test was performed for 5 min, whereas the literature recommends 10 min, and no females were introduced into the cage to increase the aggressiveness of the resident animal.
7. Comment on possible translational implications and future research directions	The study used simvastatin, a drug widely used to treat dyslipidemia, and the relationship between this drug and sleep deprivation may have potential translational implications. The use of simvastatin as a means of reducing glial reactivity may be something that can be explored by other studies and for other pathologies. We intend to increase the number of markers for astrocytes and microglia and introduce females for evaluation in this same context.
