# Supplementary material for: Simvastatin Modulates Astrocyte and Microglial Reactivity and Aggressive Behavior After Chronic Sleep Deprivation in Rats
Source: J Neurosci Res. 2026 Nov 1;104(11):e70160. doi: 10.1002/jnr.70160 (PMC13724290; doi:10.1002/jnr.70160)
Supplement: Supplementary file 2 [file JNR-104-e70160-s001.docx]

S1.Photos of modified multi-platform (MMP), with 18 acrylic platforms across the water tank used to induce sleep deprivation.

S2. Photo of doses of edible small pellets formed by a mixture of simvastatin and a powdered base for preparing whipped cream, used for the administration of the vehicle and the statins.
